# New protocols for the synthesis of 5-amino-7-(4-phenyl)-4,7-dihydro-[1,2,4]triazolo[1,5-a]pyrimidine-6-carboxylate esters using an efficient additive

**DOI:** 10.3906/kim-2005-6

**Published:** 2020-08-18

**Authors:** Nader GHAFFARI KHALIGH, Taraneh MIHANKHAH, Salam TITINCHI, Zohreh SHAHNAVAZ, Mohd RAFIE JOHAN

**Affiliations:** 1 Nanotechnology and Catalysis Research Center, Institute of Postgraduate Studies, University of Malaya, Kuala Lumpur Malaysia; 2 Department of Water and Environmental Engineering, Iran University of Science and Technology, Tehran Iran; 3 Department of Chemistry, University of the Western Cape, Cape Town South Africa

**Keywords:** Organic catalysis, Lewis base, heterocycles, three-component reactions, sustainable chemistry

## Abstract

This work introduces a new additive named 4,4’-trimethylenedipiperidine for the practical and ecofriendly preparation of ethyl 5-amino-7-(4-phenyl)-4,7-dihydro-[1,2,4]triazolo[1,5-a]pyrimidine-6-carboxylate derivatives. This chemical is commercially available and easy to handle. It also possesses a low melting point and a broad liquid range temperature, high thermal stability, and good solubility in water. Based on green chemistry principles, the reaction was performed in a) a mixture of green solvents i.e. water and ethanol (1:1 v/v) at reflux temperature, and b) the additive was liquefied at 65 °C and the reaction was conducted in the liquid state of the additive. High yields of the desired triazolo-pyrimidines were obtained under both aforementioned conditions. Our results demonstrated that this additive, containing 2 Lewis base sites and able to act as an acceptor-donor hydrogen bonding group, is a novel and efficient alternative to piperidine, owing to its unique properties such as its reduced toxicity, nonflammable nature, nonvolatile state, broad liquid range temperature, high thermal stability, and ability to be safely handled. Furthermore, this additive could be completely recovered and exhibited high recyclability without any change in its chemical structure and no significant reduction in its activity. The current methodology has several advantages: (a) it avoids the use of hazardous materials, as well as toxic, volatile, and flammable solvents, (b) it does not entail tedious processes, harsh conditions, and the multistep preparation of catalysts, (c) it uses a metal-free and noncorrosive catalyst, and (d) reduces the generation of hazardous waste and simple work-up processes. The most important result of this study is that 4,4’-trimethylenedipiperidine can be a promising alternative for toxic, volatile, and flammable base reagents in organic synthesis owing to its unique properties.

## 1. Introduction

Solvents play a vital role in both academic and industrial research, and they can influence the reaction mechanism and route, selectivity, reaction rate, type, and yield of a product. Hence, the choice of an appropriate solvent is a vital issue in organic reactions. An ideal solvent for organic reactions should be commercially available, costeffective, nontoxic, nonvolatile, biocompatible, biodegradable, recoverable, reusable, and have low risks associated with its handling and storage; furthermore, a nonflammable solvent with a high flash point and selective soluble is preferred. The environmental, technical, and economic issues related to the solvent, how it is used, and what it is made from are important factors in selecting a sustainable solvent [1]. Ionic liquids (ILs) have been widely used as solvents and/or catalysts in a variety of organic reactions. The fabrication of some ionic liquids involves tedious procedures and harsh conditions, the use of toxic and corrosive reagents or expensive reactants, as well as toxic, flammable, and volatile solvents [2]. To overcome these issues, the design and development of sustainable solvents that are clean and involve green media is an attractive and promising area in organic chemistry.

According to the principles of green chemistry, the design and development of new efficient catalysts and methods require researchers to follow several standards such as (a) using inexpensive, less toxic, nonvolatile solvents and chemicals for the fabrication of catalysts, for the separation of products and workup, or recovery of catalysts; (b) using green solvents or conducting reactions under solvent-free conditions, which results in reducing hazardous waste generation; (c) avoiding toxic and metal-containing catalysts, as well as using recyclable catalysts which promote reactions by using their catalytic amount [3,4].

Triazolo[1,5-a]pyrimidines (TPs) display a broad range of pharmaceutical and medicinal properties [5–8]. Various substituted triazolo[1,5-a]pyrimidines have been prepared through a one-pot, three-component condensation of active methylene compounds, aminotriazoles, and aldehydes using different catalytic systems [6]. The developed methods have certain drawbacks, along with several advantages. There are very often two or more preparative steps for the fabrication of the catalysts, which can raise the cost and may involve the use of toxic and volatile solvents. Some other methods require a tedious work-up, together with the washing and rinsing the products or catalysts multiple times, which leads to the generation of toxic and hazardous wastes. Hence, there is a high demand for the development of metal-free protocols, which employ nontoxic solvents, along with easily separable and recyclable catalysts.

Very recently, a series of novel 1,2,4-triazolo[1,5-a]pyrimidines were prepared using ionic liquid and porous organic polymer [9,10]. Based on the unique properties of 4,4’-trimethylenedipiperidine (TMDP) and its successful catalytic applications in some organic synthesis [11–13], we were encouraged to investigate the potential of TMDP as a catalyst or dual solvent-catalyst for the preparation of 1,2,4-triazolo[1,5-a]pyrimidines through a one-pot three-component reaction.

## 2. Results and discussion

### 2.1. Synthesis of 1,2,4-triazolo[1,5-a]pyrimidine derivatives in the presence of TMDP as catalyst or dual solvent-catalyst

Initially, the condensation of 3 model reactants, including 4-chlorobenzaldehyde (1a), 3-amino-1,2,4-triazole, and ethyl cyanoacetate was investigated in different conditions to find the optimal conditions (Table 1). The equimolar model reactants were stirred together in the water as a green solvent at room temperature or reflux condition for 2 h in the absence of a catalyst (Table 1, entries 1 and 2). A large amount of the unreacted 4-chlorobenzaldehyde (1a) was detected together with a few spots on the TLC at reflux temperature, and only substrates were detected at room temperature. The addition of a catalytic amount of TMDP caused a remarkable rise in the yield of 2a under reflux conditions (Table 1, entries 3 and 4). Regarding the limited solubility of substrates in water, the next experiments were conducted in a mixture solvent of water and ethanol (50:50% v/v), which lead to an improvement of the yield of 2a (Table 1, entry 5). The amount of TMDP was then increased two-fold, which caused a negligible rise in the yield of 2a Table 1, entry 6). These results demonstrate that this three-component reaction required a catalyst and higher temperature due to its high activation energy.

In our previous work, it was observed that TMDP has a low melting point (52.3 ◦C) and a high boiling point (332.5 ◦C) [13]. Hence, TMDP has a wider liquid range (~280 ◦C) than water and ethanol. Furthermore, TMDP has 2 Lewis base sites, and it can simultaneously act as both a hydrogen bond acceptor and donor. In the next experiments, we conducted the model reaction at 65 ◦C, and TMDP changed into its molten state; the mixture of reactants was easily stirred in the liquefied TMDP (Table 1, entries 7–9). The highest yield was observed for a length of time lasting approximately 60 min (Table 1, entry 8).

**Scheme 1:**
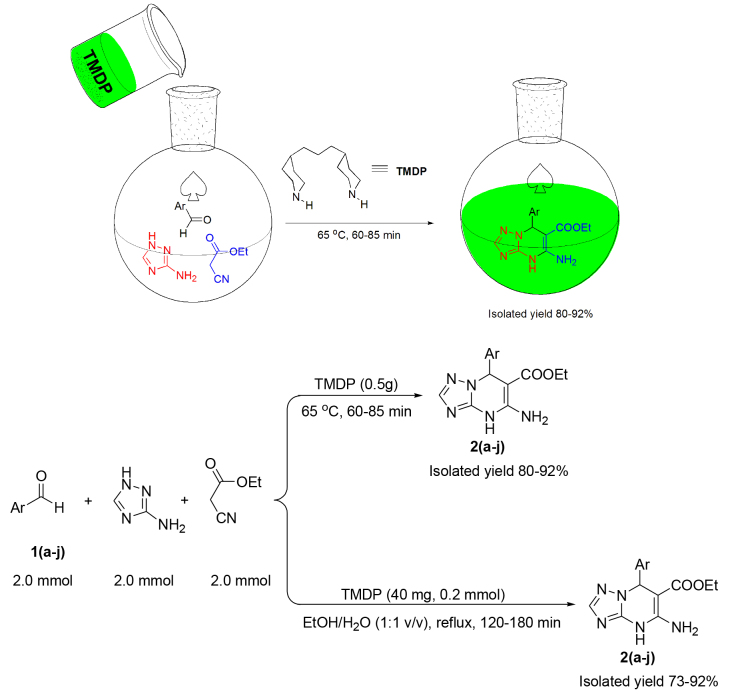
Preparation of ethyl 5-amino-7-(4-phenyl)-4,7-dihydro-[1,2,4]triazolo[1,5-a]pyrimidine-6-carboxylates using (a) molten-state TMDP (b) a solution of TMDP in a mixture of solvents ethanol/water (1:1 v/v).

We chose entries 6 and 8 in Table 1 as the optimal conditions and investigated the scope and generality of the current protocols (Scheme 1).

**Table 1:** Optimization of different parameters including TMDP loading, temperature, and stirring time for the model reaction.^a^

Entry	Amount of TMDP (mg)	Solvent	Temp. (◦C)	Time (min)	Yield (%)^b^
1	-	H_2_O	r.t.	120	-
2	-	H_2_O	Reflux	120	A few spots
3	40	H_2_O	r.t.	120	32
4	40	H_2_O	Reflux	120	70
5	40	H_2_O/EtOH (1:1 v/v)	Reflux	120	91
6	80	H_2_O/EtOH (1:1 v/v)	Reflux	120	92
7	500	Liquefied TMDP	65	120	92
8	500	Liquefied TMDP	65	60	92
9	500	Liquefied TMDP	65	40	77

^a^Reaction conditions: 4-chlorobenzaldehyde (285.0 mg, ∼2.0 mmol), ethyl cyanoacetate (244 μL, 2.0 mmol), 3-amino-1,2,4-triazole (168 mg, 2.0 mmol), solvent (2 mL). ^b^Yields after trituration of the reaction mixture in water and recrystallization from ethanol.

Benzaldehydes bearing electron-withdrawing and electron-donating substituents smoothly incorporated the corresponding triazolo[1,5-a]pyrimidines under both optimized reaction conditions. The results in Table 2 show that the effect of nature and the position of substituents in the yields and rates are negligible within the experimental error. Nevertheless, benzaldehydes bearing electron-donating groups produced lower yields after longer reaction times in comparison with electron-withdrawing substituents in the same positions (Table 2, entries 1–6,10). Moreover, the para-substituted benzaldehydes bearing the electron-donating groups exhibited lower yields than the electron-withdrawing substituents (Table 2, entries 3,5,10). Also, ortho-substituted benzaldehydes resulted in higher yields than the same substituents at the para-positions (Table 2, entries 3,5 vs. 4,6). All known products showed a melting point and 1 H NMR and 13 C NMR consistent with previous reports in the literature [9]. The 1 H NMR and 13 C NMR of 5-amino-7-(4-phenyl)-4,7-dihydro-[1,2,4]triazolo[1,5-a]pyrimidine-6-carboxylate esters 2(a–j), together with their copies, can be found in Appendix A (see Supplementary Material).

**Table 2:** The preparation of ethyl 5-amino-7-(4-phenyl)-4,7-dihydro-[1,2,4]triazolo[1,5-a]pyrimidine-6-carboxylates using TMDP as a catalyst or catalyst/solvent, respectively, under 2 optimized reaction conditions.^a^

Entry	1(a–j)	2(a–j)	Method A^b^		Method B^c^		Melting point (◦C)	
			Time (min)	Yield (%)^d^	Time (min)	Yield (%)^d^	Found	Reported [9]
1	4-Cl-C_6_H_4-_	2a	60	92	120	92	190–191	190–191
2	4-Br-C_6_H_4-_	2b	60	90	120	87	181–183	184–185
3	4-NO_2_-C_6_H_4-_	2c	60	93	100	91	196–198	197–198
4	2-NO_2_-C_6_H_4-_	2d	70	86	140	81	180–182	183–184
5	4-(CH_3_O)-C_6_H_4-_	2e	85	87	140	84	188–189	187–188
6	2-(CH_3_O)-C_6_H_4-_	2f	85	81	120	76	202–203	205–206
7	3,4-(CH_3_O)-C_6_H_3-_	2g	80	83	120	79	189–190	190–191
8	2,4,6-(CH_3_O)_3_-C_6_H_2-_	2h	85	80	150	76	206–207	206–207
9	3,4,5-(CH_3_O)_3_-C_6_H_2-_	2i	80	80	120	76	210–212	211–212
10	4-(CH_3_)_2_N-C_6_H_4-_	2j	85	80	180	73	181–182	181–182

^a^Reaction conditions: various substituted benzaldehydes 1(a–j) (0.5 mmol), ethyl cyanoacetate (61 μL, 0.5 mmol); 3-amino-1,2,4-triazole (42 mg, 0.5 mmol). ^b^Molten state of TMDP (125 mg); reaction temperature (65 ◦C). ^c^10 mol% of TMDP (10.5 mg, 0.05 mmol); a mixture of ethanol and water (0.5 mL, 1:1 v/v) at reflux temperature. ^d^Isolated yield.

The model reaction was also carried out using 20 μL (~10 mol%) and 40 μL (~20 mol%) of piperidine under optimized conditions in 2.0 mL of ethanol/H_2_O, which resulted in the desired product at 32% and 73% yields, respectively. In another experiment, 2.0 mmol of model reactants were added into 0.5 g of piperidine and stirred at 65 ◦C for 60 min, which gave 2a at a 86% yield. Although the 2 reagents, TMDP and piperidine, can be interchanged under the appropriate conditions, TMDP is preferred because of the high toxicity and unsafe handling of piperidine. Besides, there is limited piperidine availability in some institutions and/or countries due to its use as a substrate for illegal, psychotropic drugs. This can therefore cause administrative problems when purchasing piperidine [14].

Very recently, Jonnalagadda et al. fabricated a heterogeneous catalyst viz. the bismuth loaded on fluorapatite (Bi_2_O_3_/FAp), which was used for the preparation of 1,2,4-triazolo[1,5-a]pyrimidine scaffolds in ethanol at room temperature [15]. Despite high yields of the products (92–96%) within 25–35 min using 30 mg catalyst per 1 mmol benzaldehyde derivatives, the fabrication of the catalyst has drawbacks; for example, (a) there is a mixture of metal-contained Lewis acids in the catalyst composition, in which each salt can play a role in promoting the reaction, while the effect of the different cations and anions were not clarified and investigated; (b) high energy-consuming procedure due to high temperature for a long time and centrifugation; (c) the catalyst was rinsed several times with water during the catalyst preparation, which can lead to generating hazardous waste via leakage of cations and anions. Furthermore, Lewis acids are not traditional catalysts for the preparation of pharmaceutical and medicine products due to their high toxicity [16]. Also, it is well known that aldehydes bearing substituents such as methoxy, nitro-, cyano-, or heterocycle aldehydes containing nitrogen or sulphur atoms, and coordinating solvents such as water can deactivate Lewis acids by the coordination of their acidic sites [17,18]. Moreover, most Lewis acids require an activation step before application. All the above results show that the catalytic efficiency of TMDP is an efficient, safe, and ecofriendly catalyst for the synthesis of 1,2,4-triazolo[1,5-a]pyrimidines.

**Scheme 2:**
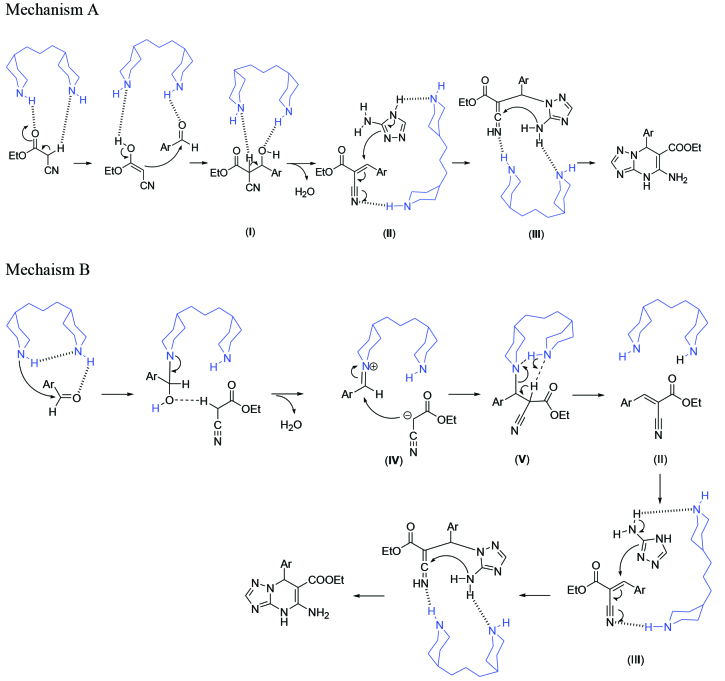
The possible schematic reaction mechanisms.

According to the mechanism of the Knoevenagel reaction in the literature, 2 possible mechanisms are illustrated in Scheme 2 [19,20]. Based on reaction mechanism A, the ethyl cyanoacetate and aldehyde are activated through hydrogen bonding and the Lewis base property of TMDP. The ethyl cyanoacetate attacks the activated benzaldehyde, which gives the intermediate (I). The dehydration of intermediate (I) gives the Knoevenagel intermediate (II). The B route involves the iminium intermediate (IV), which acts as the acceptor of the enol of ethyl cyanoacetate and gives the corresponding Knoevenagel product. After this, in the A and B routes, TMDP can activate 3-amino-1,2,4-triazole, followed by a the reaction with the Knoevenagel intermediate (II), which gives the intermediate (III). Then, the intermediate (III) produces the triazolo[1,5-a]pyrimidine through an intramolecular cyclization.

Finally, the condensation of model reactants was carried out on a large scale as an industrial application. A flask fitted with a mechanical stirrer was charged with 12.50 g of TMDP and heated up to 65 ◦C. Then, 7.24 g of 4-chlorobenzaldehyde and 4.42 g of 3-amino-1,2,4-triazole, as well as 5.43 mL of ethyl cyanoacetate (5.428 mL) were slowly added to the liquefied TMDP with continuous stirring. After 1 h, 15 mL of deionized water was added to the mixture, and the white solid was filtered on a Büchner funnel and rinsed 3 times with 5 mL of deionized water. The purification of the crude product was conducted by recrystallization from the hot ethanol (20 mL), which gave 13.11 g of 2a (82%). After removing the water from the filtrate, the recovered TMDP was reused for the mmole scale reactions.

### 2.2. Easy separation and recyclability of TMDP

The separation of TMDP and the crude products was conducted through the trituration of the reaction mixture in water and subsequent filtration. TMDP was recovered through solvent removal by a rotary evaporator in a vacuum. The results demonstrated that the recovered TMDP could be reused for 10 subsequent runs, which enabled 2a reach a range of 92–82% isolated yield. Although a 5.6-wt. % loss of TMDP was detected during 10 runs during the recovering process, ^1^H NMR analysis of retrieved TMDP showed no detectable changes in the chemical structure of TMDP after the 10th run (Figure). The leaching of TMDP onto the product was also investigated by an LC-MS analysis of 2a, which showed a negligible amount of TMDP after each run.

**Figure:**
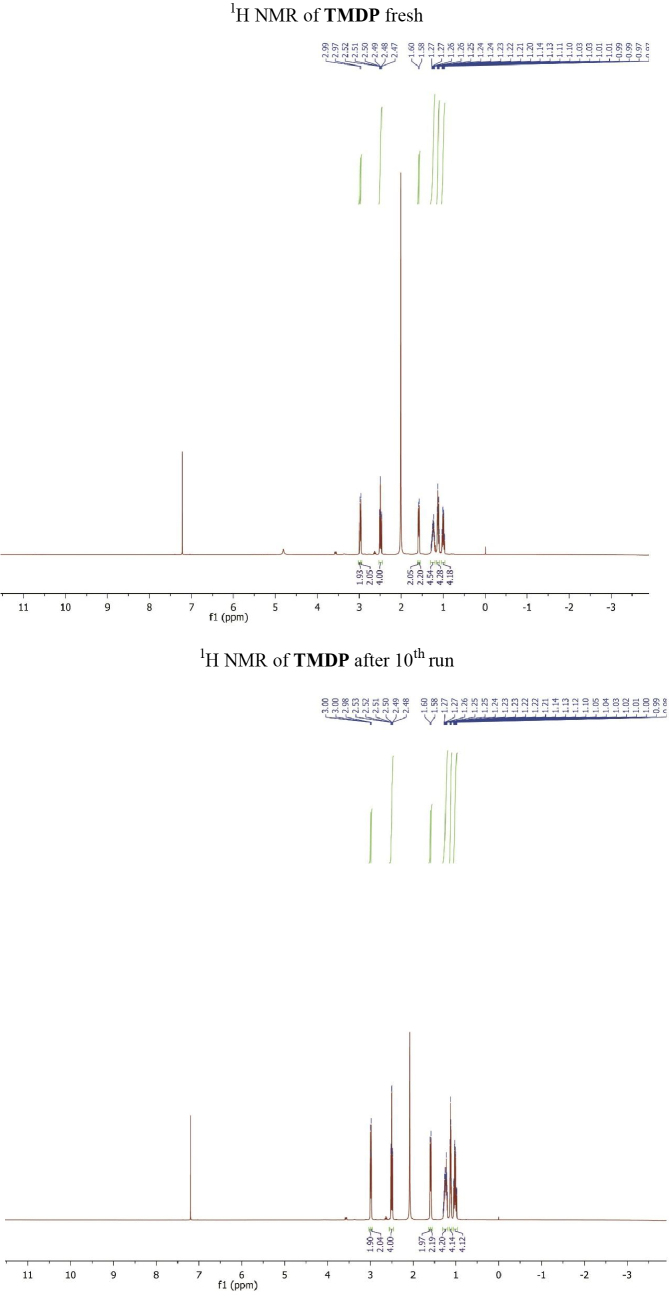
^1^H NMR of TMDP fresh (up) and ^1^H NMR of TMDP after 10^th^ run (down).

## 3. Conclusion

TMDP was employed to catalyze a one-pot three-component reaction in its molten-state as a dual solventcatalyst under mild conditions. The preparation of 1,2,4-triazolo[1,5-a]pyrimidine derivatives demonstrated the efficiency of TMDP as a high recyclable additive and dual solvent catalyst. The advantages of the current methodologies are (a) they are metal-free and mild conditions; (b) there is high yield within short reaction times; (c) there is a simple separation of the catalyst or solvent/catalyst and desired products; (d) there is minimal generation of hazardous wastes. The unique features of TMDP, such as the fact that it is commercially available, that it has a wide liquid range, that is bears Lewis base sites and hydrogen bond acceptor-donor groups, and that it has high recyclability, make it a promising catalyst for organic synthesis. In addition, TMDP can be a safe alternative for toxic, flammable, and volatile organic base catalysts.

## 4. Experimental

### 4.1. General

All chemicals were supplied by Merck, Aldrich, and Fluka chemical companies and used as purchased. The TLC and silica gel SIL G/UV 254 plates were employed to determine the purity of substrates and for reaction monitoring. The samples were charged into open capillary tubes, and their melting point was measured on a Büchi B-545 apparatus. A Bruker Avance 400 MHz instrument was used to record the ^1^H and ^13^C NMR spectra. Microanalyses were performed on a Perkin-Elmer 240-B microanalyzer.

### 4.2. Typical procedure for the preparation of ethyl 5-amino-7-(4-chlorophenyl)-4,7-dihydro-[1,2,4] triazolo[1,5-a]pyrimidine-6-carboxylate (2a) using TMDP as a catalyst in mixture of water and ethanol (1:1 v/v)

A mixture of 4-chlorobenzaldehyde (285.0 mg, 2.0 mmol), 3-amino-1,2,4-triazole (168 mg, 2.0 mmol), ethyl cyanoacetate (244 μL, 2.0 mmol), and TMPD (40 mg, ~10 mol%) was stirred in water/ethanol (2.0 mL, 1:1 v/v) for an appropriate time at reflux conditions. After completion of the reaction (monitored by TLC), the solvent was removed with a rotary evaporator under reduced pressure. Then 1.0 mL of water was added with a dropping tube, the suspension was thoroughly mixed by a stirring rod, and the additive and product were separated by simple filtration. The white solid was rinsed with a few drops of water (20 drops), which gav 612.0 mg of crude product (~95%). The crude product was dissolved in 1.0 mL of ethanol at 50 ◦C, which produced 580.0 mg of pure product (~92%) after cooling to room temperature overnight. After the additio of an appropriate amount of ethanol to the aqueous solution of TMDP, it was reused in the next run without any washing, drying, or purification.

### 4.3. Typical procedure for the synthesis of ethyl 5-amino-7-(4-chlorophenyl)-4,7-dihydro-[1,2,4] triazolo[1,5-a]pyrimidine-6-carboxylate (2a) using TMDP as dual solvent-catalyst in the molten-state

The mixture of 4-chlorobenzaldehyde (285.0 mg, 2.0 mmol), 3-amino-1,2,4-triazole (168 mg, 2.0 mmol), and ethyl cyanoacetate (244 μL, 2.0 mmol) was stirred in TMPD (0.5 g) at 65 ◦C. After completion of the reaction (monitored by TLC), the deionized water (0.5 mL) was added to the reaction mixture. The crude product and additive were separated by simple filtration. The crude product was purified by recrystallization from ethanol by the same above-mentioned procedure, which gave 583 mg of white powder (~92%). The solvent was removed from the aqueous solution of TMDP under reduced pressure with a rotary evaporator, and the recovered TMDP was reused in the next runs without any rinsing, drying, or purification.

Supplementary Materials Click here for additional data file. Supplementary data, including ^1^H NMR, ^13^ C NMR and the elemental analysis of 2(a–j), together with ^1^H NMR, and ^13^C NMR copies of 2(a–j), can be found in the Appendix A-Supplementary Material.
