# Supplementary material for: New protocols for the synthesis of 5-amino-7-(4-phenyl)-4,7-dihydro-[1,2,4]triazolo[1,5-a]pyrimidine-6-carboxylate esters using an efficient additive
Source: Turk J Chem. 2020 Aug 18;44(4):1100–9. doi: 10.3906/kim-2005-6 (PMC7751900; doi:10.3906/kim-2005-6)
Supplement: Supplementary file 1 — Supplementary Materials [file turkjchem-44-1100-sup001.pdf]

Supplementary material

^{1}HNMR and ^{13}C NMR of 2(a–j) (NMR spectra were recorded in a 400 MHz Bruker instrument, and DMSO-d_6 was used as an NMR solvent).

2a)

^{1}H NMR δ8.83 (s, 2H, NH_2), 8.41 (s, 1H, triazole-CH), 8.07 (d, J = 8.6 Hz, 2H, Ar-H), 7.66 (d, J = 8.6 Hz, 2H, Ar-H), 6.56 (s, 1H, NH), 5.65 (s, 1H, CH), 4.31 (q, J = 7.2 Hz, 2H, –(C=O)OC$\underline{H}_2$CH_3), 1.30 (t, J = 7.2 Hz, 3H, –(C=O)OCH_2C$\underline{H}_3$) ppm; ^{13}C NMR (100 MHz, DMSO-d_6) δ 161.6 (C=O), 153.6 (triazole-C), 143.1 (C5), 138.0 (triazole-CH), 132.4 (C-2' and C-6'), 130.2 (C-1'), 129.4 (C-3' and C-5'), 115.4 (C-4'), 103.2 (C-6), 62.44 (–(C=O)O$\underline{C}H_2$CH_3), 51.7 (C-7), 14.0 (–(C=O)OC$H_2\underline{C}H_3$) ppm; Anal. Calcd. For $C_{14}H_{14}ClN_5O_2$: C, 52.59; H, 4.41; N, 21.90; Found: C, 52.57; H, 4.45; N, 21.87.

2b)

^{1}H NMR δ 8.68 (s, 2H, NH_2), 8.42 (s, 1H, triazole-CH), 7.98 (d, J = 8.6 Hz, 2H, Ar-H), 7.81 (d, J = 8.6 Hz, 2H, Ar-H), 6.43 (s, 1H, NH), 5.63 (s, 1H, CH), 4.36 (q, J = 7.2 Hz, 2H, –(C=O)OC$\underline{H}_2$CH_3), 1.30 (t, J = 7.2 Hz, 3H, –(C=O)OCH_2C$\underline{H}_3$) ppm; ^{13}C NMR (100 MHz, DMSO-d_6) δ 161.6 (C=O), 153.8 (triazole-C), 140.0 (C5), 132.5 (C-2' and C-6'), 132.4 (C-3' and C-5'), 130.5 (triazole-CH), 127.1 (C-1'), 115.4 (C-4'), 103.4 (C-6), 62.4 (–(C=O)O$\underline{C}H_2$CH_3), 52.0 (C-7), 14.0 (–(C=O)OC$H_2\underline{C}H_3$) ppm; Anal. Calcd. For $C_{14}H_{14}BrN_5O_2$: C, 46.17; H, 3.87; N, 19.23; Found: C, 46.14; H, 3.91; N, 19.21.

2c)

^{1}H NMR δ 8.85 (s, 1H, triazole-CH), 8.53 (s, 2H, NH_2), 8.04 (d, J = 8.2 Hz, 2H, Ar-H), 7.63 (d, J = 8.2 Hz, 2H, Ar-H), 6.61 (s, 1H, NH), 5.64 (s, 1H, CH), 4.34 (q, J = 7.2 Hz, 2H, –(C=O)OC$\underline{H}_2$CH_3), 1.34 (t, J = 7.2 Hz, 3H, –(C=O)OCH_2C$\underline{H}_3$) ppm; ^{13}C NMR (100 MHz, DMSO-d_6) δ 163.6 (C=O), 152.9 (triazole-C), 143.2 (C5), 139.2 (triazole-CH), 132.5 (C-2' and C-6'), 130.5 (C-3' and C-5'), 128.2 (C-1'), 115.3 (C-4'), 103.2 (C-6), 62.3 (–(C=O)O$\underline{C}H_2$CH_3), 51.8 (C-7), 14.0 (–(C=O)OC$H_2\underline{C}H_3$) ppm; Anal. calcd. for $C_{14}H_{14}N_6O_4$: C, 50.91; H, 4.27; N, 25.44; Found: C, 50.88; H, 4.32; N, 25.47.

2d)

^{1}H NMR δ 8.83 (s, 1H, triazole-CH), 8.55 (s, 2H, NH_2), 8.32 (d, J = 8.2 Hz, 1H, Ar-H), 7.97-7.91 (m, 2H, Ar-H), 7.84 (t, J = 7.6 Hz, 1H, Ar-H), 6.61 (s, 1H, NH), 5.63 (s, 1H, CH), 4.35 (q, J = 7.0 Hz, 2H, –(C=O)OC$\underline{H}_2$CH_3), 1.33 (t, J = 7.0 Hz, 3H, –(C=O)OCH_2C$\underline{H}_3$) ppm; ^{13}C NMR δ 160.9 (C=O), 154.9 (triazole-C), 147.2 (C-2'), 141.2 (C5), 134.7 (triazole-CH), 132.5 (C-6'), 130.5 (C-5'), 128.2 (C-1'), 125.2 (C-4'), 114.2 (C-3'), 107.4 (C6), 62.7 (–(C=O)O$\underline{C}H_2$CH_3), 52.0 (C-7), 13.9 (–(C=O)OC$H_2\underline{C}H_3$) ppm; Anal. calcd. for $C_{14}H_{14}N_6O_4$: C, 50.91; H, 4.27; N, 25.44; Found: C, 50.86; H, 4.33; N, 25.41.

2e)

^{1}H NMR (400 MHz, DMSO-d_6) δ 8.57 (s, 2H, NH_2), 8.31 (s, 1H, triazole-CH), 8.07 (d, J = 8.8 Hz, 2H, Ar-H), 7.13 (d, J = 8.8 Hz, 2H, Ar-H), 6.43 (s, 1H, NH), 5.22 (s, 1H, CH), 4.28 (q, J = 7.0 Hz, 2H, –(C=O)OC$\underline{H}_2$CH_3), 3.85 (s, 3H, –OCH_3), 1.28 (t, J = 7.0 Hz, 3H, –(C=O)OCH_2C$\underline{H}_3$) ppm; ^{13}C NMR (100 MHz, DMSO-d_6) δ 163.5 (C=O), 162.3 (C-4'), 154.4 (triazole-C), 137.1 (C5), 133.5 (C-2' and C-6'), 123.9 (triazole-CH), 116.2 (C-1'), 114.9 (C-3' and C-5'), 98.5 (C-6), 62.0 (–(C=O)O$\underline{C}H_2$CH_3), 55.7 (O$\underline{C}H_3$), 51.7 (C-7), 14.0 (–(C=O)OC$H_2\underline{C}H_3$) ppm; Anal. calcd. for $C_{15}H_{17}N_5O_3$: C, 57.13; H, 5.43; N, 22.21; Found: C, 57.08; H, 5.47; N, 22.24.

2f)

^{1}H NMR (400 MHz, DMSO-d_6) δ 8.54 (s, 1H, triazole-CH), 8.35 (s, 2H, NH_2), 8.12 (d, J = 7.8 Hz, 1H, Ar-H), 7.64-7.59 (m, 1H, Ar-H), 7.20-7.17 (m, 1H, Ar-H), 7,13-7.09 (m, 1H, Ar-H), 6.37 (s, 1H, NH), 5.65 (s, 1H, CH), 4.30 (q, J = 7.0 Hz, 2H, –(C=O)OC$\underline{H}_2$CH_3), 3.87 (s, 3H, –OCH_3), 1.29 (t, J = 7.0 Hz, 3H, –(C=O)OC$\underline{H}_2$CH_3) ppm; ^{13}C NMR (100 MHz, DMSO-d_6) δ 161.9 (C=O), 158.8 (C-2'), 148.9 (triazole-C), 140.6 (C5), 135.5 (C-6'), 128.4 (C-4'), 120.7 (triazole-CH), 119.7 (C-1'), 115.6 (C-5'), 112.1 (C-3'), 102.1 (C-6), 62.3 (–(C=O)O$\underline{C}H_2$CH_3), 56.0 (O$\underline{C}H_3$), 51.7 (C-7), 13.9 (–(C=O)OC$H_2\underline{C}H_3$) ppm; Anal. calcd. for $C_{15}H_{17}N_5O_3$: C, 57.13; H, 5.43; N, 22.21; Found: C, 57.11; H, 5.45; N, 22.18.

2g)

^{1}H NMR δ8.45 (s, 2H, NH_2), 8.23 (s, 1H, triazole-CH), 7.72 (s, 1H, Ar-H), 7.69 (dd, J = 8.6 and 1.6 Hz, 1H, Ar-H), 7.14 (d, J = 8.4 Hz, 1H, Ar-H), 6.38 (s, 1H, NH), 5.21 (s, 1H, CH), 4.30 (q, J = 7.0 Hz, 2H, –(C=O)OC$\underline{H}_2$CH_3), 3.88 (s, 3H, –OCH_3), 3.80 (s, 3H, –OCH_3), 1.31 (t, J = 7.0 Hz, 3H, –(C=O)OC$\underline{H}_2$CH_3) ppm; ^{13}C NMR (100 MHz, DMSO-d_6) δ 162.4 (C=O), 154.6 (C-3'), 153.6 (C-4'), 148.8 (triazole-C), 131.1 (C5), 126.8 (triazole-CH), 124.0 (C-1'), 116.3 (C-6'), 113.0 (C-5'), 111.9 (C-2'), 98.6 (C-6), 62.0 (–(C=O)O$\underline{C}H_2$CH_3), 56.0 (O$\underline{C}H_3$), 55.5 (O$\underline{C}H_3$), 52.0 (C-7), 14.0 (–(C=O)OC$H_2\underline{C}H_3$) ppm; Anal. calcd. for $C_{16}H_{19}N_5O_4$: C, 55.64; H, 5.55; N, 20.28; Found: C, 55.61; H, 5.58; N, 20.24.

2h)

^{1}H NMR δ8.61 (s, 2H, NH_2), 8.26 (s, 1H, triazole-CH), 6.65 (s, 1H, NH), 6.31 (s, 2H, Ar-H), 5.71 (s, 1H, CH), 4.25 (q, J = 7.0 Hz, 2H, –(C=O)OC$\underline{H}_2$CH_3), 3.86 (s, 3H, –OCH_3), 3.84 (s, 6H, –OCH_3), 1.26 (t, J = 7.0 Hz, 3H, –(C=O)OC$\underline{H}_2$CH_3) ppm; ^{13}C NMR δ 165.8 (C=O), 163.1 (C-4'), 160.8 (C-2' and C-6'), 146.7 (triazole-C), 140.0 (C5), 115.6 (triazole-CH), 103.2 (C-3' and C-5'), 102.1 (C-1'), 91.0 (C-6), 61.8 (–(C=O)O$\underline{C}H_2$CH_3), 55.8 (OCH_3 at the *para*-position), 55.6 (OCH_3 at the *ortho*-position), 51.4 (C-7), 14.0 (–(C=O)OC$H_2\underline{C}H_3$) ppm; Anal. calcd. for $C_{17}H_{21}N_5O_5$: C, 54.39; H, 5.64; N, 18.66; Found: C, 54.42; H, 5.59; N, 18.61.

2i)

^{1}H NMR δ 8.62 (s, 2H, NH_2), 8.27 (s, 1H, triazole-CH), 6.66 (s, 1H, NH), 6.43 (s, 2H, Ar-H), 5.61 (s, 1H, CH), 4.29 (q, J = 7.0 Hz, 2H, –(C=O)OC$\underline{H}_2$CH_3), 3.82 (s, 3H, –OCH_3), 3.80 (s, 6H, –OCH_3), 1.28 (t, J = 7.0 Hz, 3H, –(C=O)OC$\underline{H}_2$CH_3) ppm; ^{13}C NMR δ 167.4 (C=O), 165.8 (C-4'), 160.8 (C-3' and C-5'), 141.7 (triazole-C), 130.0 (C5), 105.6 (triazole-CH), 103.3 (C-1'), 102.1 (C-2' and C-6'), 91.0 (C-6), 62.0 (–(C=O)O$\underline{C}H_2$CH_3), 55.8 (OCH_3 at the *para*-position), 55.6 (OCH_3 at the *meta*-position), 51.4 (C-7), 14.0 (–(C=O)OC$H_2\underline{C}H_3$) ppm; Anal. calcd. for $C_{17}H_{21}N_5O_5$: C, 54.39; H, 5.64; N, 18.66; Found: C, 54.41; H, 5.57; N, 18.69.

2j)

^{1}H NMR δ 8.36 (s, 2H, NH_2) 8.06 (s, 1H, triazole-CH), 7.92 (d, J = 9.0 Hz, 2H, Ar-H), 6.81 (d, J = 9.0 Hz, 2H, Ar-H), 6.27 (s, 1H, NH), 5.21 (s, 1H, CH), 4.24 (q, J = 7.0 Hz, 2H, –(C=O)OC$\underline{H}_2$CH_3), 3.05 (s, 6H, –N(CH_3)$_2$), 1.25 (t, J = 7.0 Hz, 3H, –(C=O)OC$\underline{H}_2$CH_3) ppm; ^{13}C NMR δ 163.4 (C=O), 154.1 (C-4'), 153.7 (triazole-C), 137.2 (C5), 133.7 (C-2' and C-6'), 118.3 (triazole-CH), 117.5 (C-1'), 111.6 (C-3' and C-5'), 92.0 (C-6), 61.4 (–(C=O)O$\underline{C}H_2$CH_3), 52.6 (C-7), 40.1 (–N($\underline{C}H_3$)$_2$), 14.1 (–(C=O)OC$H_2\underline{C}H_3$) ppm; Anal. calcd. for $C_{17}H_{21}N_5O_5$: C, 58.52; H, 6.14; N, 25.59; Found: C, 58.48; H, 6.19; N, 25.56.

^{1}H and ^{13}C NMR copies of 2(a–j) were recorded with a 400 MHz Bruker instrument in DMSO-d_6.
